# Low-Illumination Image Enhancement Algorithm Based on Improved Multi-Scale Retinex and ABC Algorithm Optimization

**DOI:** 10.3389/fbioe.2022.865820

**Published:** 2022-04-11

**Authors:** Ying Sun, Zichen Zhao, Du Jiang, Xiliang Tong, Bo Tao, Guozhang Jiang, Jianyi Kong, Juntong Yun, Ying Liu, Xin Liu, Guojun Zhao, Zifan Fang

**Affiliations:** ^1^ Key Laboratory of Metallurgical Equipment and Control Technology of Ministry of Education, Wuhan University of Science and Technology, Wuhan, China; ^2^ Research Center for Biomimetic Robot and Intelligent Measurement and Control, Wuhan University of Science and Technology, Wuhan, China; ^3^ Hubei Key Laboratory of Mechanical Transmission and Manufacturing Engineering, Wuhan University of Science and Technology, Wuhan, China; ^4^ Precision Manufacturing Research Institute, Wuhan University of Science and Technology, Wuhan, China; ^5^ Hubei Key Laboratory of Hydroelectric Machinery Design and Maintenance, China Three Gorges University, Yichang, China

**Keywords:** multi-scale retinex, weighted guided image filtering, ABC algorithm, bilateral gamma function, image enhancement

## Abstract

In order to solve the problems of poor image quality, loss of detail information and excessive brightness enhancement during image enhancement in low light environment, we propose a low-light image enhancement algorithm based on improved multi-scale Retinex and Artificial Bee Colony (ABC) algorithm optimization in this paper. First of all, the algorithm makes two copies of the original image, afterwards, the irradiation component of the original image is obtained by used the structure extraction from texture via relative total variation for the first image, and combines it with the multi-scale Retinex algorithm to obtain the reflection component of the original image, which are simultaneously enhanced using histogram equalization, bilateral gamma function correction and bilateral filtering. In the next part, the second image is enhanced by histogram equalization and edge-preserving with Weighted Guided Image Filtering (WGIF). Finally, the weight-optimized image fusion is performed by ABC algorithm. The mean values of Information Entropy (IE), Average Gradient (AG) and Standard Deviation (SD) of the enhanced images are respectively 7.7878, 7.5560 and 67.0154, and the improvement compared to original image is respectively 2.4916, 5.8599 and 52.7553. The results of experiment show that the algorithm proposed in this paper improves the light loss problem in the image enhancement process, enhances the image sharpness, highlights the image details, restores the color of the image, and also reduces image noise with good edge preservation which enables a better visual perception of the image.

## Introduction

The vast majority of information acquired by humans comes from vision. Images, as the main carrier of visual information, play an important role in three-dimensional reconstruction, medical detection, automatic driving, target detection and recognition and other aspects of perception (Li B. et al., 2019; Wang et al., 2019; Yu et al., 2019; Huang et al., 2021; Liu et al., 2022a; Tao et al., 2022a; Yun et al., 2022a; Bai et al., 2022). With the rapid development of optical and computer technology, equipment for image acquisition are constantly updated, and images often contain numerous valuable information waiting to be discovered and accessed by humans (Jiang et al., 2019a; Huang et al., 2020; Hao et al., 2021a; Cheng and Li, 2021). However, due to the influence of light, weather and imaging equipment, the captured images are often dark, noisy, poorly contrasted and partially obliterated in detail in real life (Sun et al., 2020a; Tan et al., 2020; Wang et al., 2020). This kind of image makes the area of interest difficult to identify, thus reducing the quality of image and the visual effect of the human eyes (Jiang et al., 2019b; Hu et al., 2019), and also causes great inconvenience for the extraction and analysis of image information, generating considerable difficulty for computers and other vision devices to carry out normal target detection and recognition (Su and Jung, 2018; Sun et al., 2020b; Cheng et al., 2020; Luo et al., 2020; Hao et al., 2021b). Therefore, it is necessary to enhance the low-light images through image enhancement technology (Jiang et al., 2019c; Sun et al., 2020c), so as to highlight the detailed features of the original images, improve contrast, reduce noise, make the original blurred and low recognition images clear, improve the recognition and interpretation of images comparatively, and satisfy the requirements of certain specific occasions (Tao et al., 2017; Ma et al., 2020; Jiang et al., 2021a; Tao et al., 2021; Liu et al., 2022b). Metaheuristic algorithms have great advantages for multi-objective problem solving and parameter optimization (Li et al., 2020a; Yu et al., 2020; Chen et al., 2021a; Liu X. et al., 2021; Wu et al., 2022; Xu et al., 2022; Zhang et al., 2022; Zhao et al., 2022), Methods of Multiple Subject Clustering and Subject Extraction as well as, K-means clustering methods, steady-state analysis methods, numerical simulation techniques quantification and regression methods are also widely used in data processing (Li et al., 2020b; Sun et al., 2020d; Chen et al., 2022). Artificial Bee Colony (ABC) is an optimization method proposed to imitate the honey harvesting behavior of bee colony, which is a specific application of cluster intelligence idea. The main feature is that ABC requires no special information about the problem, but only needs to compare the advantages and disadvantages of the problem (Li C. et al., 2019; He et al., 2019; Duan et al., 2021), and through the individual local optimization-seeking behavior of each worker bee, the global optimum value will eventually emerge in the population, which has a fast convergence speed (Chen et al., 2021b; Yun et al., 2022b).

In response to the above problems, considering this advantage of ABC, this paper proposes a low-illumination image enhancement algorithm based on improved multi-scale Retinex and ABC optimization. Based on Retinex theory and image layering processing, this algorithm improves and optimizes the multi-scale Retinex algorithm with the structure extraction from texture via relative total variation, and replicates the original image to obtain the main feature layer and the compensation layer. In the image fusion process, the ABC algorithm is used to optimize the fusion weight factors of each layer and select the optimal solution to realize the processing enhancement of low-illumination images. Finally, the effectiveness of the algorithm in this paper is verified by conducting experiments on the LOLdataset dataset.

The other parts of this paper as follows: *Related Work* gives an overview of image enhancement methods in low illumination and Artificial Bee Colony algorithms; *Basic Theory* describes the basic theory of Retinex; *The Algorithm Proposed in This Paper* proposes a low illumination image enhancement algorithm based on improved multiscale Retinex and ABC optimization; *Experiments and Results Analysis* conducts verification experiments which compares with the traditional Retinex algorithm and the method proposed in this paper and the results were analyzed by Friedman test and Wilcoxon signed rank test; and the conclusions of this paper are summarized in *Conclusion*.

## Related Work

Image enhancement algorithms are grouped into two main categories: spatial domain and frequency domain image enhancement algorithms (Vijayalakshmi et al., 2020). The methods of spatial domain enhancement mainly include histogram equalization (Tan and Isa, 2019) and Retinex algorithm, etc.

Histogram Equalization (HE) achieves the enhancement of image contrast by adjusting the pixel grayscale of the original image and mapping the image grayscale to more gray levels to make it evenly distributed, but often the noise of image processed by HE is also enhanced and the details are lost (Nithyananda et al., 2016); The Retinex image enhancement method proposed by Land E H (Land, 1964) combines well with the visual properties of the human eye, especially in low-illumination enhancement, and which performs well overall compared to other conventional methods. Based on the Retinex theory, Jobson D J et al.(Jobson et al., 1997) proposed the Single-Scale Retinex (SSR) algorithm, which can get better contrast and detail features by estimating the illumination map, but this algorithm can cause detail loss in image enhancement. Researchers subsequently proposed Multi-Scale Retinex (MSR), the image enhanced by this algorithm will have certain problems of color bias, and there will still be local unbalanced enhancement and “halo” phenomenon (Wang et al., 2021). Therefore, Rahman Z et al. (Rahman et al., 2004) proposed the Multi-Scale Retinex with Color Restoration (MSRCR), and the “halo” and color problems have been improved. The application of convolutional neural networks to deep learning has led to improved enhancement and recognition, but the difficulties in the construction of the network and the collection of data sets for training make this method difficult to implement (Liu et al., 2021b; Sun et al., 2021; Weng et al., 2021; Yang et al., 2021; Tao et al., 2022b; Liu et al., 2022c). Based on the Retinex algorithm, Wang D et al. (Wang et al., 2017) used Fast Guided Image Filtering (FGIF) to evaluate the irradiation component of the original image, combined with bilateral gamma correction to adjust and optimize the image, which preserved the details and colors of the image to some extent, but the overall visual brightness was not high. Zhai H et al. (Zhai et al., 2021) proposed an improved Retinex with multi-image fusion algorithm to operate and fuse three copies of images separately, and the images processed by this algorithm achieved some improvement in brightness and contrast, but the overall still had noise and some details lost.

The frequency domain enhancement methods mainly include Fourier transform, wavelet transform, Kalman filtering and image pyramid, etc (Li et al., 2019c; Li et al., 2019d; Huang et al., 2019; Chang et al., 2020; Tian et al., 2020; Liu et al., 2021c). This kind of algorithm can effectively enhance the structural features of the image, but the target details of the image which are enhanced by these methods are still blurred. The image layering enhancement method proposed by researchers in recent years has led to the application of improved low-light image enhancement methods based on this principle more and more widely (Liao et al., 2020; Long and He, 2020). The enhancement of image layer decomposes the input image into base layer and detail layer components, and then processes the enhancement of the two layers separately, and finally selects the appropriate weighting factor for image fusion. Commonly used edge-preserving filters are bilateral filtering, Guided Image Filtering (GIF), Fast Guided Image Filtering (Singh and Kumar, 2018), etc. Since GIF uses the same linear model and weight factors for each region of the image, it is difficult to adapt to the differences in texture features between different regions of the image. In order to resolve this problem of GIF, Li Z et al.(Li et al., 2014) proposed a Weighted Guided Image Filtering (WGIF) based on local variance, which constructs an adaptive weighting factor based on traditional guided filtering, which not only improves the edge-preserving ability but also reduces the “halo artifacts” caused by image enhancement.

Inspired by the honey harvesting behavior of bee colonies, Karaboga (Karaboga, 2005) proposed a novel global optimization algorithm based on swarm intelligence, Artificial Bee Colony (ABC), in 2005. Since its introduction, the ABC algorithm has attracted the attention of many scholars and has been analyzed comparatively. Karaboga et al. (Karaboga and Basyurk, 2008) analyze the performance of ABC compared with other intelligent algorithms under multidimensional and multimodal numerical problems and the effect of the scale of the ABC control parameters taken. Karaboga et al. (Karaboga and Akay, 2009) were the first to perform a detailed and comprehensive performance analysis of ABC by testing it against 50 numerical benchmark functions and comparing it with other well-known evolutionary algorithms such as Genetic Algorithms (GA), Particle Swarm Optimization (PSO), Differential Evolution Algorithm (DE), and Ant Colony Optimization (ACO). Akay et al. (Akay and Karaboga, 2009) analyzed the effect of parameter variation on ABC performance. Singh et al. (Singh, 2009) proposed an artificial bee colony algorithm for solving minimum spanning tree and verified the superiority of this algorithm for solving such problems. Ozurk et al. (Ozurk and Karaboga, 2011) proposed a hybrid method of artificial bee colony algorithm and Levenberg-Marquardts for the training of neural networks. Karaboga et al. (Karaboga and Gorkemli, 2014) modified the new nectar search formula to find the best nectar source near the exploited nectar source (set a certain radius value) to be exploited in order to improve the local merit-seeking ability of the swarm algorithm.

## Basic Theory

### Fundamentals of Retinex

**FIGURE 1:**
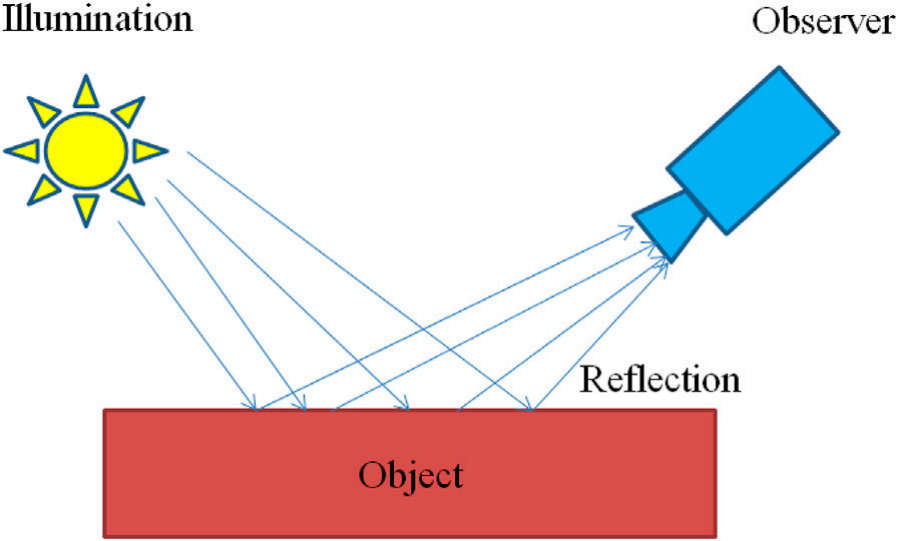
Retinex schematic.

Retinex is a common method of image enhancement based on scientific experiments and scientific analysis, which is proposed by Edwin.H.Land in 1963 (Land and McCann, 1971). In this theory, two factors determine the color of an object being observed, as shown in Figure 1, namely the reflective properties of the object and the intensity of the light around the them, but according to the theory of color constancy, it is known that the inherent properties of the object are not affected by light, and the ability of the object to reflect different light waves determines the color of the object to a large extent (Zhang et al., 2018).

This theory shows that the color of the substance is consistent and depends on its ability to reflect wavelengths, which is independent of the absolute value of the intensity of the reflected light, in addition to being unaffected by non-uniform illumination, and is consistent, so Retinex is based on color consistency. While traditional nonlinear and linear only enhance one type of feature of the object, this theory can be adjusted in terms of dynamic range compression, edge enhancement and color invariance, enabling adaptive image enhancement.

The Retinex method assumes that the original image is obtained by multiplying the reflected image and the illuminated image, which can be expressed as

$$I_{\left(x,y\right)}=R_{\left(x,y\right)}\cdot L_{\left(x,y\right)}$$ (1)

In Eq. 1, *I*_*(x,y)*_ is the original image, *R*_*(x,y)*_ is the reflection component with the image details of the target object, *L*_*(x,y)*_ is the irradiation component with the intensity information of the surrounding light.

In order to reduce the computational complexity in the traditional Retinex theory, the complexity of the algorithm is usually simplified by taking logarithms on both sides with a base of 10 of Eq. 1 and converting the multiplication and division operations in the real domain to the addition and subtraction operations in the logarithmic domain. The conversion results are as follows:

$$\mathrm{lg}I_{\left(x,y\right)}=\mathrm{lg}R_{\left(x,y\right)}+\mathrm{lg}L_{\left(x,y\right)}$$ (2)

### Traditional Retinex Algorithm

The SSR method uses a Gaussian kernel function as the central surround function to obtain the illumination component by convolving with the original image and then subtracting it to obtain the reflection component in the logarithmic domain.

The specific expressions are as follows:

$$r_{\left(x,y\right)}=\mathrm{lg}\left[R_{\left(x,y\right)}\right]=\mathrm{lg}\left[\frac{I_{\left(x,y\right)}}{L_{\left(x,y\right)}}\right]=\mathrm{lg}I_{\left(x,y\right)}-\mathrm{lg}L_{\left(x,y\right)}$$ (3)

$$L_{\left(x,y\right)}=G_{\left(x,y\right)}*I_{\left(x,y\right)}$$ (4)

$$G_{\left(x,y\right)}=\delta e^{-\left(x^{2}+y^{2}\right)/\sigma^{2}}$$ (5)

$$\iint G_{\left(x,y\right)}dxdy=1$$ (6)

In Eqs 4, 5, $G_{\left(x,y\right)}$ denotes the center surround function - Gaussian kernel function, $L_{\left(x,y\right)}$ is obtained by convolving $G_{\left(x,y\right)}$ with $I_{\left(x,y\right)}$ . σ is the Gaussian surround scale parameter, and is the only adjustable parameter in SSR. When σ is small, it can retain better image details, but the color is easily distorted; When σ is larger, better image color can be preserved, but the details of image easily loss (Parihar and Singh, 2018; Jiang et al., 2021b).

In order to maintain high image fidelity and compression of the dynamic range of the image, researchers proposed the Multi-Scale Retinex (MSR) method on the basis of SSR(Peiyu et al., 2020), The MSR algorithm uses multiple Gaussian wrap-around scales for weighted summation, The specific expressions are as follows:

$$r_{\left(x,y\right)}=\sum_{k=1}^{K}\omega_{k}\left[\mathrm{lg}I_{\left(x,y\right)}-\mathrm{lg}\left(G_{k_{\left(x,y\right)}}*I_{\left(x,y\right)}\right)\right]$$ (7)

In Eqs. 7, K is the number of Gaussian center surround functions. When K = 1, MSR degenerates to SSR. $\omega_{k}$ is the weighting factor under different Gaussian surround scales, and in order to ensure the advantages of both high, medium and low scales of SSR to be considered, K is usually taken as three and $\omega_{1}=\omega_{2}=\omega_{3}=1/3$ .

Considering the color bias problem of SSR and MSR, the researchers developed the MSRCR (Weifeng and Dongxue, 2020), MSRCR adds a color recovery factor to MSR, which is used to adjust the color ratio of the channels, The specific expressions are as follows:

$$r_{MSRCR}^{\left(x,y\right)}=C_{i}\left(x,y\right)r_{MSRi}^{\left(x,y\right)}$$ (8)

$$C_{i}\left(x,y\right)=\beta \left\{\mathrm{lg}\left[\alpha I_{i}\left(x,y\right)\right]-\mathrm{lg}\left[\sum_{j=1}^{N}I_{j}\left(x,y\right)\right]\right\}$$ (9)

In Eq. 9, β is the gain constant; α is the nonlinear intensity control parameter; $I_{i}\left(x,y\right)$ denotes the image of the *i*th channel. $\sum_{j=1}^{N}I_{j}\left(x,y\right)$ denotes the sum of pixels in this channel. After processing the image by MSRCR algorithm, the pixel values usually appear negative. So the color balance is achieved by linear mapping and adding overflow judgment to achieve the desired effect.

## The Algorithm Proposed in This Paper

**FIGURE 2:**
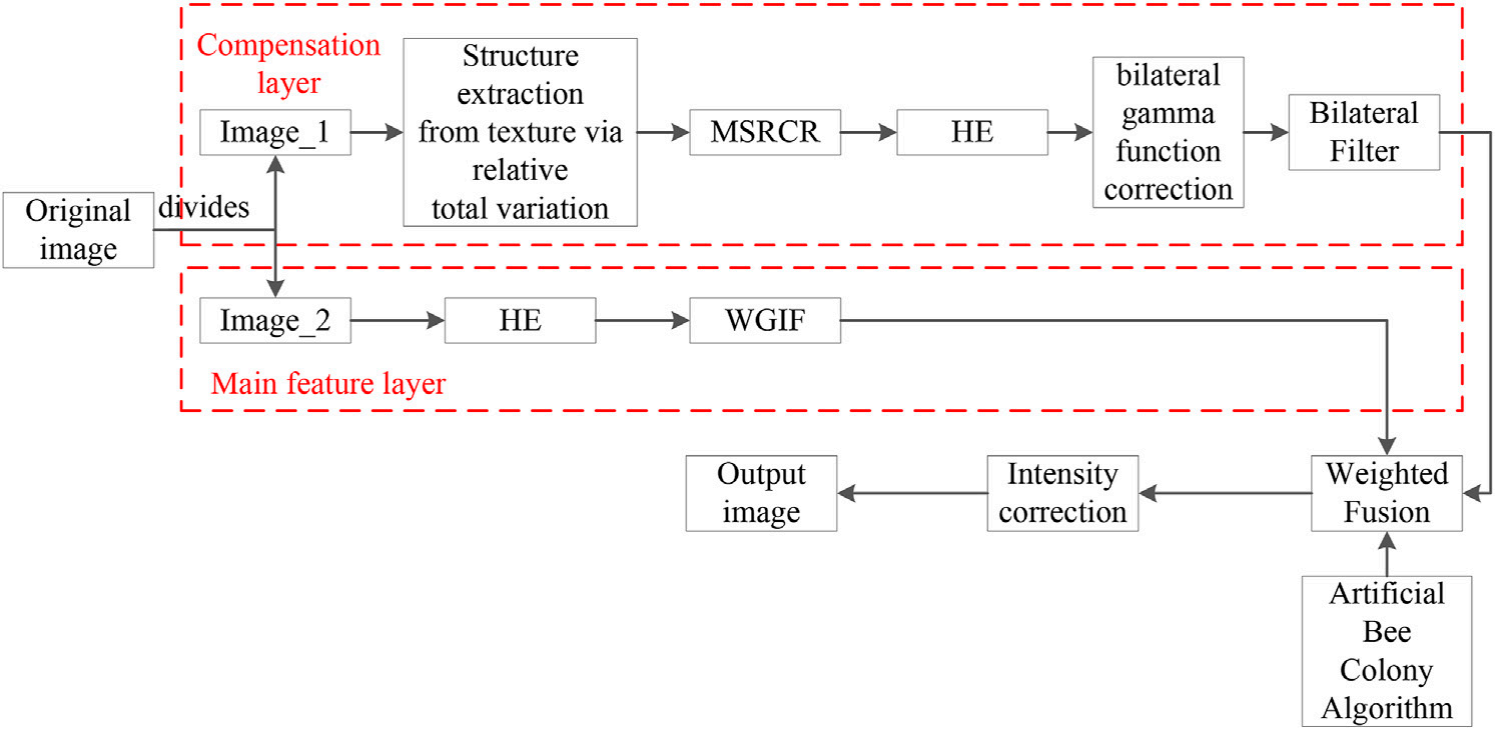
Flowchart of the algorithm in this paper.

The low-illumination image enhancement algorithm based on improved multi-scale Retinex and ABC optimization, which is proposed in this paper, divides the image equivalently into a main feature layer and a compensation layer. For the main feature layer firstly, HE is used for image enhancement, and WGIF is selected for edge-preserving noise reduction. For the compensation layer, the irradiated component of the original image is first obtained by used the structure extraction from texture via relative total variation, and then the original image is processed with the MSRCR algorithm to obtain the reflected component for color recovery, and Histogram Equalization, bilateral gamma function correction, and edge-preserving filtering are applied to it. Finally, the main feature layer and the compensation layer are fused by optimal parameters, and the optimal parameters are obtained by adaptive processing correction with an ABC algorithm to achieve image enhancement under low illumination. The flow chart of the algorithm in this paper is shown in Figure 2.

### Main Feature Layer

#### Weighted Guided Image Filtering

Guided Image Filter is a filtering method proposed by He K et al. (He et al., 2012), which is an image smoothing filter based on a local linear model. The basic idea of guided image filter is to assume that the output image is linearly related to the bootstrap image within a local window $\omega_{k}$ . A guided image is used to generate weights to derive a linear model for each pixel, and thus the input image is processed. The mathematical model expression is as follows:

$$O_{i}=a_{k}G_{i}+b_{k},\forall i\in \omega_{k}$$ (10)

To find the linear coefficients in Eq. 10, the cost function is introduced as follows:

$$E_{\left(a_{k},b_{k}\right)}=\sum_{i\in \omega_{k}}\left[{\left(a_{k}G_{i}+b_{k}-I_{i}\right)}^{2}+\varepsilon a_{k}^{2}\right]$$ (11)

Using least squares to minimize the value of the cost function $E_{\left(a_{k},b_{k}\right)}$ , the linear coefficients are obtained as:

$$a_{k}=\frac{1/\left|\omega \right|\sum_{i\in \omega_{k}}G_{i}I_{i}-\mu_{k}I_{k}}{\sigma_{k}^{2}+\varepsilon }$$ (12)

$$b_{k}=\bar{I_{k}}-a_{k}\mu_{k}$$ (13)

In Eqs. 10, 11, 12, O is the output image, G is the guide image, and I is the input image; $a_{k}$ , $b_{k}$ are the linear coefficients of the local window $\omega_{k}$ ; ε is the regularization coefficient to prevent the linear coefficient $a_{k}$ from being too large, and the larger the value of ε is, the more obvious the smoothing effect is when the input image is used as the guide image. $\mu_{k}$ denotes the mean value of G within $\omega_{k}$ , $\sigma_{k}$ denotes the standard deviation of G within $\omega_{k}$ , |ω| is the total number of pixel blocks within the local window $\omega_{k}$ and $\bar{I_{k}}$ is the mean value of the input image within the window $\omega_{k}$ .

Since a pixel point in the output image can be derived by linear coefficients in different windows, the following expression can be obtained:

$$O_{i}=\bar{a_{k}}G_{i}+\bar{b_{k}},\forall i\in \omega_{k}$$ (14)

GIF uses a uniform regularization factor ε for each region of the image, and larger regularization factors produce a “halo” phenomenon in the edge regions of the image. In view of this problem, WGIF achieves adaptive adjustment of the regularization coefficients by introducing a weighting factor $\Gamma_{G}$ . In this way, adaptive adjustment of the linear coefficients is obtained, thus achieving adaptivity to each region of the image and improving the filtering effect. The weighting factor $\Gamma_{G}$ and the new linear coefficient $a_{k}$ are as follows:

$$\Gamma_{G}=\frac{1}{N}\sum_{I^{\prime }=1}^{N}\frac{\sigma_{G,1}^{2}\left(I^{\prime }\right)+\gamma }{\sigma_{G,1}^{2}\left(I\right)+\gamma }$$ (15)

$$a_{k}=\frac{1/\left|\omega \right|\sum_{i\in \omega_{k}}G_{i}I_{i}-\mu_{k}I_{k}}{\sigma_{k}^{2}+\varepsilon /\Gamma_{G_{\left(i\right)}}}$$ (16)

In Eqs. 15, 16, $\sigma_{G,1}^{2}\left(I^{\prime }\right)$ is the variance of the guide image with respect to $\Omega_{1}\left(I^{\prime }\right)$ , where $\Omega_{1}\left(I^{\prime }\right)$ denotes a 3×3 window centered at $I^{\prime }$ and r=1 ; γ is the regularization factor, taken as ${\left(0.001\times L\right)}^{2}$ , L is the dynamic range of the image (Li et al., 2014).

**FIGURE 3:**
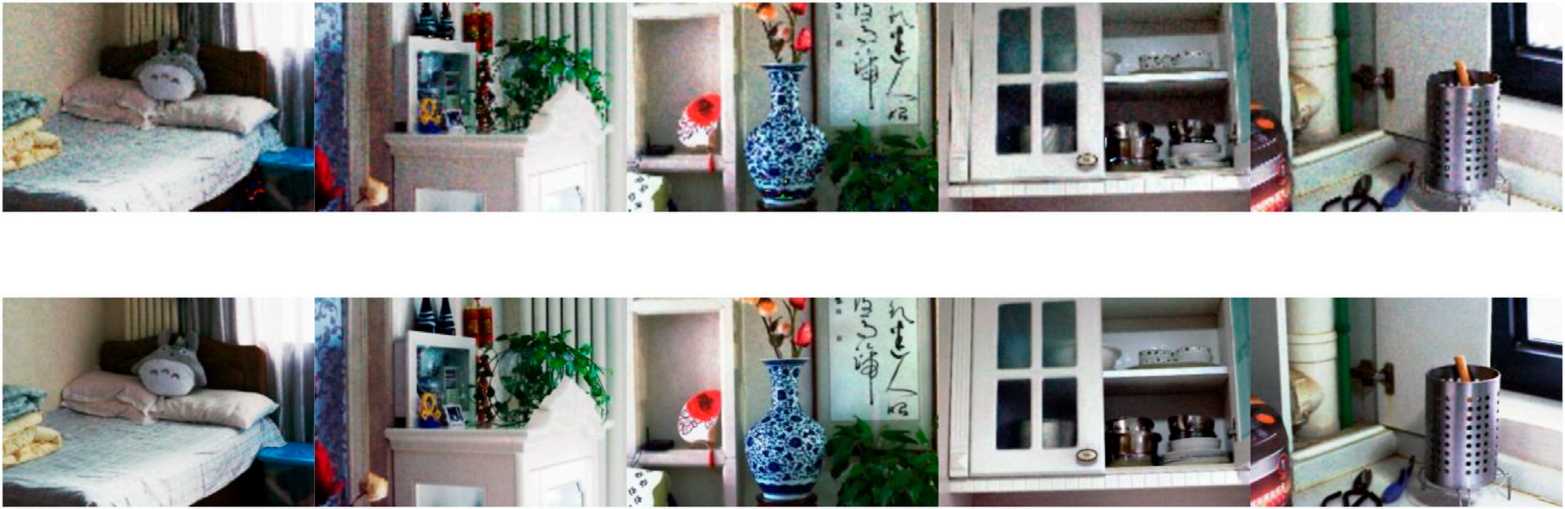
The first row is the image obtained after FGIF processing; The second row is the image obtained after WGIF processing.

A comparison of the results processed by WGIF and FGIF is shown in Figure 3. As it can be seen in Figure 3, the FGIF-processed images still have some noise, while the results after WGIF processing are well improved in this aspect.

#### Obtaining the Main Feature Layer

**FIGURE 4:**
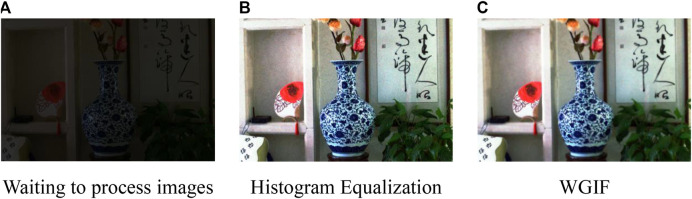
The results of main feature layer obtains. **(A)** Waiting to process images **(B)** Histogram Equalization **(C)** WGIF.

HE is used for image enhancement and WGIF is selected for edge-preserving noise reduction. The results obtained from each step are shown in Figure 4. From this figure, it can be seen that the image obtained by HE has been improved compared with the original image, but in this process, the noise in the image is also extracted and amplified. Some of the details and noise in the image are filtered out by the process of WGIF, and the “halo” phenomenon and the “artifacts” caused by the gradient inversion are avoided, because WGIF takes into account the texture differences between regions in the image.

### Compensation Layer

#### Structure Extraction From Texture via Relative Total Variation

As can be seen from 2.2, the traditional Retinex algorithm uses a Gaussian filter kernel function to convolve with the original image, and after eliminating the filtered irradiated component, the reflected component is used as the enhancement result, but the estimation of Gaussian filter at the edge of the image is prone to bias, and thus the “halo” phenomenon occurs, which undoubtedly This will undoubtedly lead to unnatural enhancement results due to the lack of illumination. To address this problem, this paper uses the structure extraction from texture via relative total variation in obtaining the irradiation component of the compensation layer, which was proposed by Xu L et al. (Xu et al., 2012) in 2012, to better preserve the main edge information of the image and thus reduce the “halo” phenomenon in the edge information-rich region. The model of the method is as follows:

$$S_{P}=\arg\min{\sum_{P}\left(S_{P}-I_{P}\right)}^{2}+\lambda \left[\frac{D_{x}\left(p\right)}{L_{x}\left(p\right)+\varepsilon }+\frac{D_{y}\left(p\right)}{L_{y}\left(p\right)+\varepsilon }\right]$$ (17)

$$D_{x}\left(p\right)=\sum_{q\in R\left(p\right)}g_{p,q}\cdot \left|{\left(\partial xS\right)}_{q}\right|$$ (18)

$$D_{y}\left(p\right)=\sum_{q\in R\left(p\right)}g_{p,q}\cdot \left|{\left(\partial yS\right)}_{q}\right|$$ (19)

$$L_{x}\left(p\right)=\left|\sum_{q\in R\left(p\right)}g_{p,q}\cdot {\left(\partial xS\right)}_{q}\right|$$ (20)

$$L_{y}\left(p\right)=\left|\sum_{q\in R\left(p\right)}g_{p,q}\cdot {\left(\partial yS\right)}_{q}\right|$$ (21)

$$g_{p,q}\propto e^{-\frac{{\left(x_{p}-x_{q}\right)}^{2}+{\left(y_{p}-y_{q}\right)}^{2}}{2\sigma^{2}}}$$ (22)

In Eqs 17 and 18, 19, 20 and 21, *S* is the output image, *p* is the pixel index, λ is the weighting factor to adjust the degree of smoothing of the image, and the larger the value of λ is, the smoother the image is; ε is a positive number close to zero and is used to prevent the denominator from being zero; $D_{x}\left(p\right)$ and $D_{y}\left(p\right)$ are respectively the variation functions of pixel *p* in the *x* and *y* directions, and R(p) is the window centered on *p*. $L_{x}\left(p\right)$ and $L_{y}\left(p\right)$ are the intrinsic functions within the window, respectively. The parameter σ is the texture suppression factor, and the larger the value of σ is, the stronger the texture suppression effect is.

**FIGURE 5:**
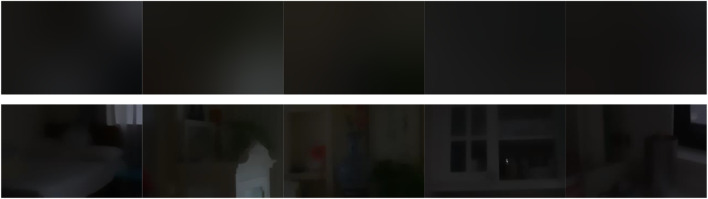
The first row is the irradiation component obtained by Gaussian kernel function; the second row is the irradiation component obtained by the structure extraction from texture via relative total variation method.

In order to demonstrate the advantages of this method from a practical point of view, the images in the LOLdataset were taken for the structure extraction from texture via relative total variation and convolution operations with the Gaussian kernel function in the traditional Retinex algorithm to obtain the irradiation components, respectively, and the results obtained by the two methods are shown in Figure 5. Meanwhile, Information Entropy and Standard Deviation were used to assess their quality, and the results are shown in Tables 1 and 2. From Figure 5, it can be seen that the structure extraction from texture via relative total variation method preserves the irradiation component better, and at the same time, it is known from the correlation evaluation function that the IE and SD of this method are greater than those of the Gaussian kernel function convolution method of the traditional Retinex algorithm, which proves that the structure extraction from texture via relative total variation method is better in preserving the image information in the acquisition of the irradiation component.

**TABLE 1:** Evaluation of IE for five sets of images.

	1	2	3	4	5
Gaussian kernel function	4.3554	4.5457	4.0200	3.2400	2.9727
This method	5.3596	5.0936	4.7888	4.7038	4.7922

**TABLE 2:** Evaluation of SD for five sets of images.

	1	2	3	4	5
Gaussian kernel function	6.7948	10.8175	5.7387	2.7720	2.7177
This method	16.5436	17.9734	8.8170	8.1452	9.0849

#### Obtaining of Compensation Layers

**FIGURE 6:**
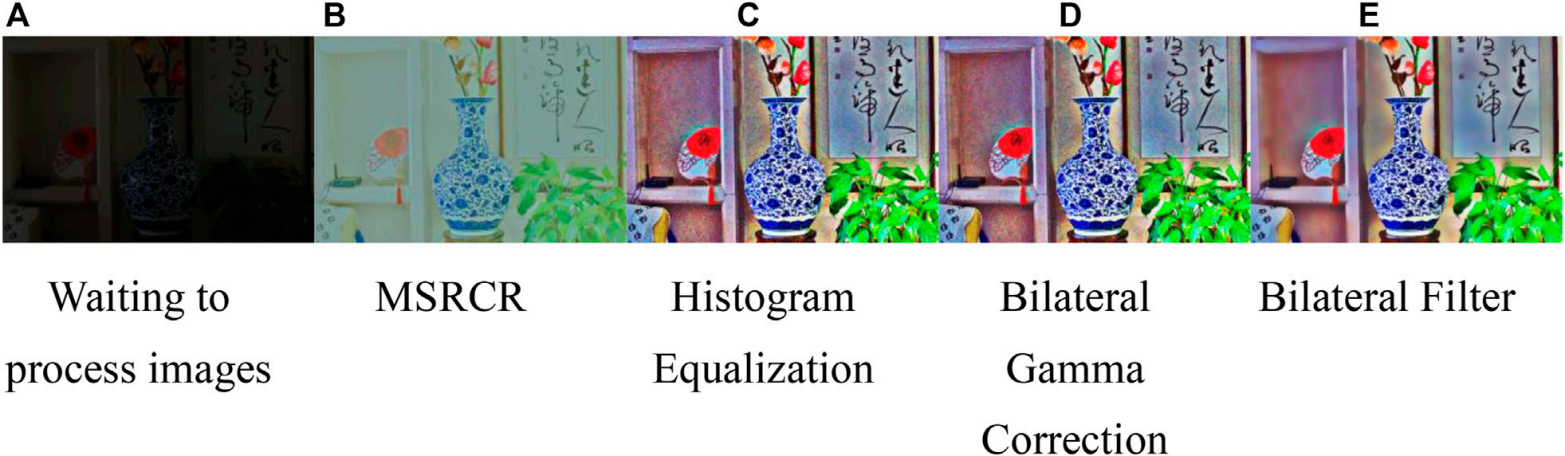
The results of compensation layers obtains.**(A)** Waiting to process images **(B)** MSRCR **(C)** Histogram Equalization **(D)** Bilateral Gamma Correction **(E)** Bilateral Filter.

For the original image, a duplication layer is performed to obtain the image to be processed, and the structure extraction from texture via relative total variation is selected to obtain the irradiation component, and combined with the principle of Retinex and color recovery to obtain the reflection component, at the same time, histogram equalization, bilateral gamma correction and bilateral filtering are performed. The results obtained from each step are shown in Figure 6. As can be seen from the figure, the image content is basically recovered by the MSRCR algorithm processing, but the image saturation is not enough to restore the real scene in comparison. After HE method, the color was recovered to some extent, but the obtained image shows that the light and dark transition areas are not effective. Therefore, this paper used the improved bilateral gamma function for processing (Wang et al., 2021). The mathematical expression of the traditional gamma function is as follows:

$$\left\{ \begin{array}{l} O_{d}\left(x,y\right)=I{\left(x,y\right)}^{\gamma }\\ O_{b}\left(x,y\right)=1-{\left(1-I\left(x,y\right)\right)}^{\gamma }\\ O\left(x,y\right)=\frac{\left[O_{d}\left(x,y\right)+O_{b}\left(x,y\right)\right]}{2} \end{array}\right.$$ (23)

In Eq. 23, I is the input image to be processed, O is the output image, and γ is a constant between (0,1) to control the enhancement performance of the image. $O_{d}$ is the convex function corrected for the dark region and $O_{b}$ is the convex function corrected for the bright region.

Since the traditional bilateral gamma function can only be mechanically enhanced, to address this problem and considering the distribution characteristics of the illumination function, the mathematical expression of the scholars’ improved bilateral gamma function is as follows:

$$\left\{ \begin{array}{l} O_{d}\left(x,y\right)=F{\left(x,y\right)}^{\gamma }\\ O_{b}\left(x,y\right)=1-{\left(1-F\left(x,y\right)\right)}^{\gamma }\\ O\left(x,y\right)=\alpha O_{d}\left(x,y\right)+\left(1-\alpha \right)O_{b}\left(x,y\right) \end{array}\right.$$ (24)

In Eq. 24, The value of γ is taken as $\gamma =0.5^{[I\left(x,y\right)-m]/m}$ ; *m* is the pixel average of the illuminated image; The adjustment parameter α takes the value of $\alpha =\left\{ \begin{array}{l} 1,I\left(x,y\right)\leq 128\\ 0,I\left(x,y\right)>128 \end{array}\right.$ .

Hence an improved bilateral gamma function is used for adaptive correction of the luminance transition region; Finally, bilateral filtering is used for edge-preserving and noise-reducing to obtain the final compensation layer.

### Image Fusion

#### Selection of the Fitness Function

Through the above processing flow, the main feature layer and compensation layer are finally obtained, and the corresponding fusion is performed at the end of the proposed method in this paper, where an image evaluation system is established and three evaluation indexes are introduced: Information Entropy, Standard Deviation and Average Gradient.

The Standard Deviation (SD) reflects the magnitude of the dispersion of the image pixels. The larger the standard deviation, the greater the dynamic range of the image and the more gradation levels. The formula to calculate SD is as follows:

$$SD=\sqrt{\frac{1}{W\times H}\sum_{i=1}^{W}\sum_{j=1}^{H}{\left(P_{\left(i,j\right)}-\mu \right)}^{2}}$$ (25)

In Equ. 25, W is the width of the input image and H is the height of the input image.

The Average Gradient (AG) represents the variation of small details in the multidimensional direction of the image. The larger the AG, the sharper the image detail, and the greater the sense of hierarchy. The formula to calculate AG is as follows:

$$AG=\frac{\sum_{i=1}^{W}\sum_{j=1}^{H}\sqrt{[{\left(\partial f/\partial x\right)}^{2}+{\left(\partial f/\partial y\right)}^{2}]/2}}{W\times H}$$ (26)

The information entropy (IE) of image is a metric used to measure the amount of information in an image. The greater the IE, the more informative and detailed the image is, and the higher the image quality. The formula to calculate IE is as follows:

$$IE=-\sum_{i=0}^{R}P\left(x\right)\log_{2}P\left(x\right)$$ (27)

In Eq. 27, *R* is the image pixel gray level, usually R = 2^8^-1, and *P(x)* is the probability that the image will appear at a point in the image when the gray value *x* is at that point.

On the concept of multi-objective optimization (Li et al., 2019e; Liao et al., 2021; Xiao et al., 2021; Liu et al., 2022d; Yun et al., 2022c), The IE, AG and SD are weighted together and balanced by using an equal proportional overlay, showing that IE, AG and SD are equally important in image evaluation. The mathematical expression of the fitness function obtained is as follows:

$$Fitness=\frac{1}{3}\left(SD+IE+AG\right)$$ (28)

**FIGURE 7:**
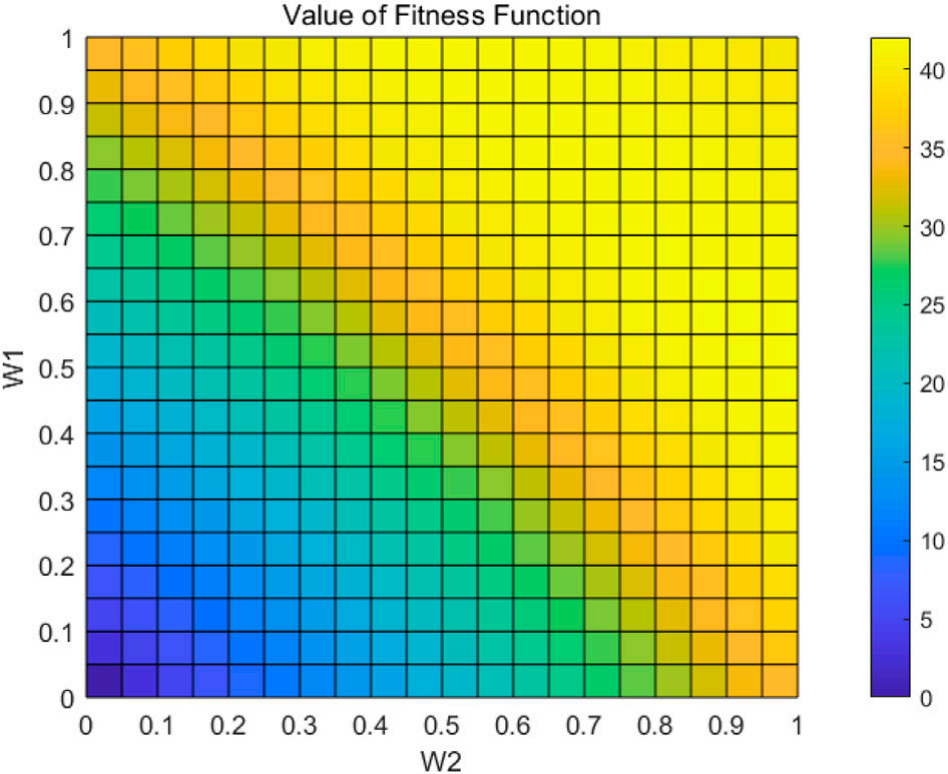
Value of fitness function with different weights.

The values of the fitness function under different weights are obtained by applying different weights to the main feature layer and the compensation layer for image weighting fusion, as shown in Figure 7. It is clear from this figure that the value of the fitness function varies with different weights and that the maximum value should be generated in $\omega_{1}\in \left[0.1,1\right],\omega_{2}\in \left[0.2,1\right]$ . To determine the optimal weights, an adaptive optimization evaluation system needs to be constructed.

Traditional nonlinear optimization algorithms update the objective solution by certain rules of derivatives, such as Gradient Descent, Newton’s Method and Quasi-Newton Methods. When solving multi-objective nonlinear optimization problems, it is difficult to satisfy the requirements because of the computational complexity of following the defined methodological steps for optimization The convergence of the Gradient Descent is slowed down when it approaches a minimal value, and requires several iterations; Newton’s method is second-order convergence, which is fast, but each step requires solving the inverse matrix of the Hessian matrix of the objective function, which is computationally complicated.

The metaheuristic algorithm models the optimization problem based on the laws of biological activity and natural physical phenomena. According to the laws of natural evolution, the natural evolution-based metaheuristic algorithm uses the previous experience of the population in solving the problem, and selects the methods that have worked well so that the target individuals are optimized in the iterations, and finally arrives at the best solution. Considering the computational complexity of the objective function and this feature of the metaheuristic algorithm, the artificial bee colony algorithm is chosen for the objective optimization.

#### Artificial Bee Colony Algorithm

Inspired by the honey harvesting behavior of bee colonies, Karaboga (2005) proposed a novel global optimization algorithm based on swarm intelligence, Artificial Bee Colony (ABC), in 2005. The bionic principle is that bees perform different activities during nectar collection according to their respective division of labor, and achieve sharing and exchange of colony information to find the best nectar source. In ABC, the entire population is divided into three types of bees, namely, employed bees, scout bees and follower bees. When a employed bee finds a honey source, it will share it with a follower bee with a certain probability; a scout bee does not follow any other bee and looks for the honey source alone, and when it finds it, it will become a employed bee to recruit a follower bee; when a follower bee is recruited by multiple employed bees, it will choose one of the many leaders to follow until the honey source is finished.

Determination of the initial location of the nectar source:

$$x_{id}=L_{d}+rand\left(0,1\right)\left(U_{d}-L_{d}\right)$$ (29)

In Eq. 29, rand(0,1) is a random number that follows a uniform distribution over the interval; $L_{d}$ and $U_{d}$ denote the upper and lower bounds of the traversal.

Leading the bee search for new nectar sources:

$$x_{id}^{new}=x_{id}+\alpha \cdot \lambda \left(x_{id}-x_{jd}\right),i\neq j$$ (30)

In Eq. 30, λ is a [-1,1] uniformly distributed random number that determines the degree of perturbation; α is the acceleration coefficient, which is usually taken as 1.

Probability of follower bees selecting the employed bee:

$$p_{i}=\frac{fitness_{i}}{\sum_{i=1}^{n}fitness_{i}}$$ (31)

Scout bees searching for new nectar sources:

$$x_{i}=\{\begin{array}{cc} L_{d}+rand\left(0,1\right)\left(U_{d}-L_{d}\right) & n\geq T\\ x_{i} & n<T \end{array}$$ (32)

**FIGURE 8:**
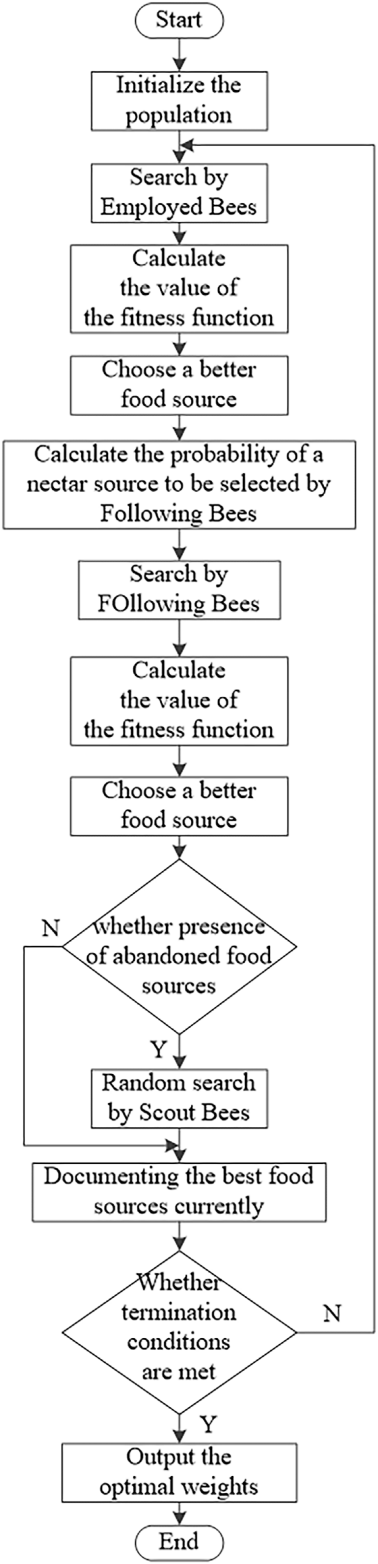
Flowchart of artificial bee colony algorithm.

During the search for the nectar source, if it has not been updated to a better one after n iterations of the search reach the threshold T, then the source is abandoned. The scout bee then finds a new nectar source again. The flow chart of the artificial bee colony algorithm is shown in Figure 8.

**FIGURE 9:**
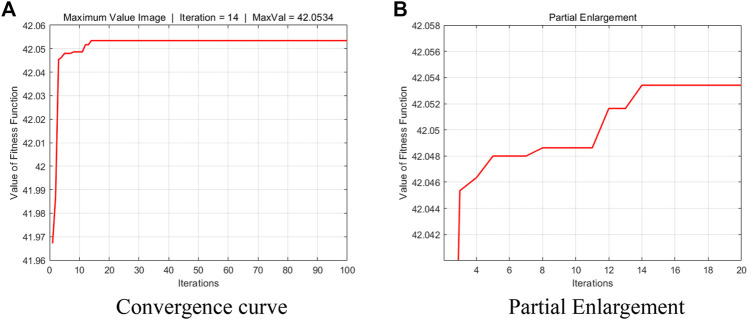
Convergence curve of artificial bee colony algorithm. **(A)** Convergence curve **(B)** Partial Enlargement.

The above fitness function is selected and iteratively optimized by the artificial bee colony algorithm, each parameter is set to the number of variables is 2, max-iter is 100, n-pop is 45, and the maximum number of honey source mining is 90. The convergence curve of the optimal weight parameter is shown in Figure 9.The convergence curves of the optimal weight parameters by iterative optimization of the artificial bee colony algorithm by selecting the above fitness function are shown in Figure 8. Considering that the optimization algorithm is to obtain the minimum value, the results are inverted, and it can be seen from Figure 9 that the maximum value is 42.0534 under this fitness function, and convergence is completed at 14 times.

## Experiments and Results Analysis

The computer used in this experiment was a 64-bit Win10 operating system; CPU为Intel(R)Core(TM) i5-6300HQ at2.30GHz; GPU is NVIDIA 960M with 2G GPU memory; RAM is 8 GB; All algorithms in this paper were run on MATLAB 2021b and Python3.7 on the PyCharm platform, and statistical analysis of the results using IBM SPSS Statistics 26.

**FIGURE 10:**
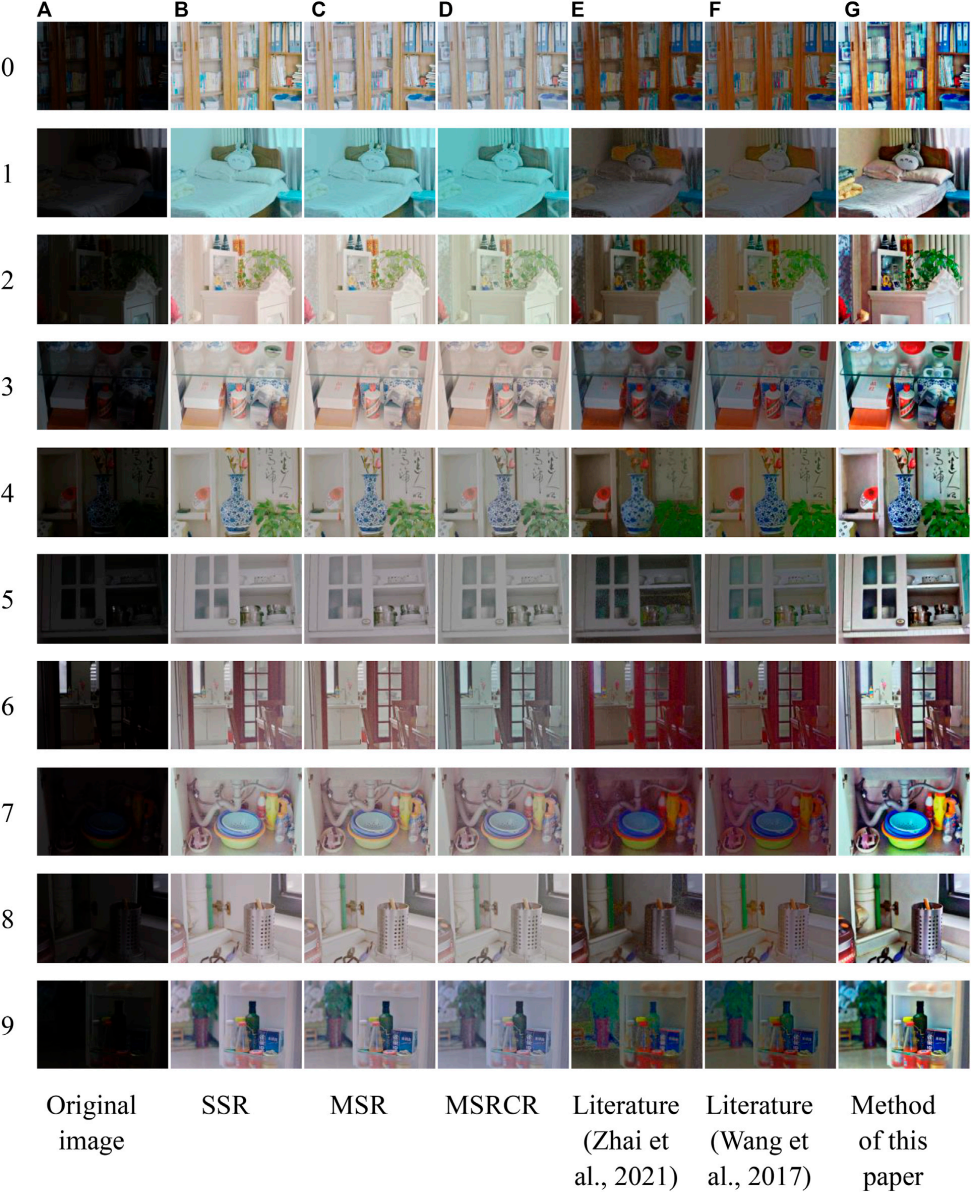
Low-illumination image processing results under different algorithms. **(A)** Original image **(B)** SSR **(C)** MSR **(D)** MSRCR **(E)** Literature (Zhai et al., 2021) **(F)** Literature (Wang et al., 2017) **(G)** Method of this paper.

The images used in the experiments are all from the LOLdataset dataset, and 200 low-illumination images are randomly selected and tested one by one by the algorithm, and the representative images are selected for comparison of processing effects. The algorithm proposed in this paper is compared with SSR algorithm, MSR algorithm, MSRCR algorithm, literature (Zhai et al., 2021), and literature (Wang et al., 2017) algorithms, where the Gaussian surround scale parameters of SSR algorithm are set to 100; the Gaussian surround scale parameters of MSR algorithm are set to 15, 80, 250; the Gaussian surround scale parameters of MSRCR algorithm are set to 15, 80, 250, *α* = 125, *β* = 46; literature (Zhai et al., 2021) and literature (Wang et al., 2017) are built according to the content of the paper respectively, and the algorithm is restored as much as possible. In this paper, the image enhancement results under different methods are analyzed by subjective evaluation and objective evaluation, and the processing results of each method are shown in Figure 10.

### Subjective Evaluation

It can be shown from Figure 10 that the brightness of the image after processing by SSR and MSR algorithms is improved compared with the original image, but the color retention effect is poor, the image is whitish and the color loss is serious. MSRCR ensure the brightness improvement comparing to the former methods, the color is also restored to some extent, but the color reproduction is not high and there is loss of details. The processing results of literature (Zhai et al., 2021) are in better color reproduction, but general brightness enhancement, part of the detailed information not effectively enhanced is still annihilated in the dark areas of the image, specifically in the end of the bed in Figures 10–e-1, the shadow of the cabinet in the lower left corner of 10-(e)-4 and the cabinet in the middle of the image of 10-(e)-8, Meanwhile, it can be seen from the image that a small amount of noise still exists in part of the location, specifically in the edge of Figures 10–e-1 and the glass of 10-(e)-5; The processing result of the literature (Wang et al., 2017) makes the brightness of the image get some improvement, basically no noise and relatively good color retention, but the image is not strong in the sense of hierarchy, specifically in the bed sheet in Figures 10–f-1 and the cabinet in 10-(f)-4. Meanwhile, it can be seen from the image that the processing images of this method have some detail loss, specifically in the restored shadows in Figures 10–f-4 and 10-(f)-8.

The enhanced image obtained by the algorithm in this paper has higher color fidelity, more prominent details, better structural information effect, and more consistent with the visual perception of human eyes in overall comparison.

### Objective Evaluation

Subjective evaluation is susceptible to interference from other factors and varies from person to person. In order to have a better comparison of the image quality of the enhancement results under different methods and to ensure the reliability of the experiments, Standard Deviation, Information Entropy and Average Gradient are used as evaluation metrics in this paper. The Standard Deviation reflects the magnitude of the dispersion of the image pixels, the greater the Standard Deviation, the greater the dynamic range of the image; Information Entropy is a metric used to measure the amount of information in an image, the higher the Information Entropy, the more information in the image. The Average Gradient represents the variation of small details in the multidimensional direction of the image, the larger the Average Gradient, the stronger the image hierarchy. The evaluation results of low-illumination image enhancement with different algorithms are shown in Tables 3–5.

**TABLE 3:** SD of low-illumination image enhancement with different algorithms.

Images	Original Image	SSR	MSR	MSRCR	Literature (Zhai et al., 2021)	Literature (Wang et al., 2017)	Method of This Paper
0	12.355	40.235	36.9085	30.6476	30.6476	33.0507	66.2931
1	17.6841	42.4476	40.1372	41.9368	41.9368	20.6165	67.0082
2	19.3482	31.1282	29.6073	26.5534	26.5534	24.7074	64.7167
3	18.1363	35.9767	31.5507	31.9188	31.9188	27.1054	67.4371
4	11.8475	32.8313	34.4849	27.4205	27.4205	27.8992	64.7396
5	10.7188	23.7767	20.9313	19.8918	19.8918	16.3517	65.5351
6	29.1959	39.6458	37.9548	36.3184	36.3184	32.8236	66.2019
7	9.4808	33.8743	28.9689	27.185	27.185	23.2276	65.4500
8	11.0124	31.6604	27.7723	25.6905	25.6905	20.4735	64.5207
9	9.2594	40.2099	35.3906	32.5299	32.5299	23.9898	65.1388

**TABLE 4:** IE of low-illumination image enhancement with different algorithms.

Images	Original Image	SSR	MSR	MSRCR	Literature (Zhai et al., 2021)	Literature (Wang et al., 2017)	Method of This Paper
0	5.3467	6.8912	6.7853	6.6234	6.5506	6.3645	7.7812
1	5.5689	6.7260	6.5138	6.4478	6.4425	5.7044	7.8505
2	5.5679	6.4388	6.2581	6.2191	6.6514	5.9694	7.7768
3	5.9126	6.8552	6.6134	6.6453	6.7434	6.3292	7.8113
4	5.2654	6.3994	6.2402	6.2048	6.4494	5.9828	7.7944
5	5.1787	6.2986	6.0710	6.0089	6.5579	5.5448	7.8458
6	5.3815	7.0954	6.9754	6.9340	6.6517	6.6910	7.6741
7	4.7559	6.9159	6.5923	6.5707	6.5038	6.0132	7.7403
8	5.0932	6.5647	6.3507	6.2511	6.6169	5.7033	7.7761
9	4.9813	6.9424	6.7524	6.6036	6.4098	6.0896	7.7995

**TABLE 5:** AG of low-illumination image enhancement with different algorithms.

Images	Original Image	SSR	MSR	MSRCR	Literature (Zhai et al., 2021)	Literature (Wang et al., 2017)	Method of This Paper
0	2.3568	8.7104	8.8584	7.2668	5.8000	6.8025	8.875
1	1.4647	5.0681	5.2774	4.6985	4.1230	4.7034	6.9498
2	1.7148	4.9633	5.2429	4.7734	3.2228	4.6958	7.8268
3	1.7484	4.8379	4.8057	4.5739	3.8150	4.6851	6.0248
4	2.0373	6.7586	6.8922	6.1064	4.4768	5.9570	8.7011
5	1.5059	3.7788	3.7817	3.5405	4.8769	4.9080	6.9163
6	1.9125	7.2986	7.7301	8.7049	5.1720	7.4135	9.1211
7	1.3312	7.0205	6.9178	6.3306	5.1602	5.7015	7.0233
8	1.3559	4.8460	4.9382	4.4152	4.4778	5.5069	6.9878
9	1.2038	6.5178	6.4573	6.0921	6.3476	6.5793	6.8033

Statistical analysis is taken for the data in Tables 3–5. The Friedman test is used to analyze the variability of the results of experiments, and the Wilcoxon sign ranked test method is used to analyze the advantages of the proposed method in this paper with other methods.

The Friedman test is a statistical test for the chi-squaredness of multiple correlated samples, which was proposed by M. Friedman in 1973. The Friedman test requires the following requirements to be met: 1. sequential level data; 2. three or more groups; 3. relevant groups; And 4. a random sample of values from the collocation. Obviously, the data in Tables 3–5 all satisfy the requirements.

Under the Friedman test, the following hypothesis is set:

H_0_: No difference between the six methods compared.

H_1_: There are differences in the six methods of comparison.

The data is imported into SPSS software for analysis, and the results were obtained as shown in Tables 6 and 7.

**TABLE 6:** The mean of Rank at Evaluation Indexes.

	SD	IE	AG
Original_image	1.00	1.00	1.00
SSR	5.80	5.60	4.70
MSR	4.70	4.40	5.20
MSRCR	3.50	3.60	3.40
Literature_Zhai	3.50	4.30	2.50
Literature_Wang	2.50	2.10	4.20
Ours	7.00	7.00	7.00

**TABLE 7:** Friedman test statistics at Evaluation Indexes.

	SD	IE	AG
Number of cases	10	10	10
χ	52.457	52.671	48.386
Degree of freedom	6	6	6
Asymptotic Significance	0.000	0.000	0.000

The Wilcoxon Signed Rank Test was proposed by F. Wilcoxon in 1945. In the Wilcoxon Signed Rank Test, it takes the rank of the absolute value of the difference between the observation and the central position of the null hypothesis and sums them separately according to different signs as its test statistic.

Under the Wilcoxon Signed Rank Test, it can be seen from Tables 3–5 that the method of this paper is numerically greater than the other algorithms, so the following hypothesis is set:

H_0_: The images enhanced by ours did not differ from the other methods.

H_1_: The images enhanced by ours differ from the other methods.

The data is imported into SPSS software for analysis, and the results of the data were obtained as shown in Table 8 and 9.

**TABLE 8:** Rank.

		Number of Cases	The Mean of Rank	The Sum of Rank
Ours - SSR	Ours < SSR	0	0.00	0.00
	Ours > SSR	10	5.50	55.00
	Ours = SSR	0		
	Total	10		
Ours - MSR	Ours < MSR	0	0.00	0.00
	Ours > MSR	10	5.50	55.00
	Ours = MSR	0		
	Total	10		
Ours - MSRCR	Ours < MSRCR	0	0.00	0.00
	Ours > MSRCR	10	5.50	55.00
	Ours = MSRCR	0		
	Total	10		
Ours - Literature_Zhai	Ours < Literature_Zhai	0	0.00	0.00
	Ours > Literature_Zhai	10	5.50	55.00
	Ours = Literature_Zhai	0		
	Total	10		
Ours - Literature_Wang	Ours < Literature_Wang	0	0.00	0.00
	Ours > Literature_Wang	10	5.50	55.00
	Ours = Literature_Wang	0		
	Total	10		

**TABLE 9:** Wilcoxon signed rank test.

	Ours - SSR	Ours - MSR	Ours - MSRCR	Ours - Literature_Zhai	Ours - Literature_Wang
Z (Based on negative rank)	−2.803	−2.803	−2.803	−2.803	−2.803
Asymptotic Significance (Bilateral)	0.005	0.005	0.005	0.005	0.005

From the data in Tables 3–5, it can be seen that the algorithm in this paper achieves a large improvement in SD, IE and AG, which is significantly better than the other five algorithms, Meanwhile, after Friedman test, it can be seen from Table 6 and 7 that asymptotic significance is less than 0.001 in all three evaluation metrics, so the original hypothesis is rejected and this data is extremely different in statistics; After Wilcoxon Signed Rank Test, it can be seen from Table 8 and 9 that the bilateral asymptotic significance is less than 0.01 for all three evaluation metrics, so the original hypothesis is rejected and the method of this paper is effective, which is differ from the other methods. This shows that the images enhanced by the algorithm in this paper have increased brightness, richer details, less image distortion and better image quality, thus verifying the effectiveness of the algorithm proposed in this paper.

## Conclusion

For the problems of poor image quality and loss of detail information in the process of low-illumination image enhancement, a low-illumination image enhancement algorithm is proposed in this paper, which is based on improved multi-scale Retinex and ABC optimization. Duplicate layering the original image, the main feature layer is processed by HE and WGIF, to enable image brightness enhancement, color restoration and noise elimination, and avoid the generation of gradient inversion artifacts; The structure extraction from texture via relative total variation method is performed on the compensation layer to estimate the irradiation component, and combined with bilateral gamma correction and other methods to avoid the occurrence of halo phenomenon; Finally, the Artificial Bee Colony algorithm is used to optimize the parameters for weighted fusion. The experimental results verify the rationality of the algorithm in this paper, and which achieves better results in both subjective and objective evaluations by comparing with other five methods.

## Data Availability

The original contributions presented in the study are included in the article/supplementary material, further inquiries can be directed to the corresponding authors.
